# Caregivers´ qualitative insights on trust, resilience and vaccination attitudes shaping child health in conflict-affected Northeast Nigeria

**DOI:** 10.1186/s13031-026-00753-w

**Published:** 2026-02-28

**Authors:** Liliana Abreu, Pia Schrage, Gbadebo Collins Adeyanju, Rabiu Ibrahim Jalo, Aisha Aliyu Abulfathi, Musa Muhammad Bello, Aminatu Ayaba Kwaku, Muhammad Ibrahim Jalo, Ahmad Mahmud, Max Schaub

**Affiliations:** 1 Department of Politics and Public Administration, University of Konstanz, Konstanz, Germany; 2 Media and Communication Science and Centre for Empirical Research in Economics and Behavioral Science (CEREB), University of Erfurt, Erfurt, Germany; 3 Bayero University & Aminu Kano Teaching Hospital, Kano, Nigeria; 4 Aminu Kano Teaching Hospital, Kano, Nigeria; 5 University of Maiduguri, Maiduguri, Nigeria; 6 University of Maiduguri Teaching Hospital, Maiduguri, Nigeria; 7 Women and Children Hospital Damaturu, Damaturu, Nigeria; 8 Modibbo Adama University, Yola, Nigeria; 9 Modibbo Adama University Teaching Hospital, Yola, Nigeria; 10 WZB Berlin Social Science Center, Berlin, & University of Hamburg, Hamburg, Germany

**Keywords:** Health-seeking behavior, Armed conflict, Vaccine hesitancy, Northeast Nigeria, Trust, Child mortality, Qualitative research

## Abstract

**Background:**

Northeast Nigeria, particularly the conflict-affected Borno, Yobe and Adamawa (BAY) states, has some of the highest under-five mortality rates in the world. Armed conflict, damaged health infrastructure and systemic poverty have significantly reduced access to healthcare. This study explores how health-seeking behaviour among caregivers of children under five intersects with trust in health systems, exposure to violence and vaccine hesitancy.

**Methods:**

A qualitative study was conducted using nine focus group discussions (FGDs) with 72 caregivers living in conflict-affected communities in the BAY states. Participants were purposively selected, and discussions explored barriers to accessing healthcare, trust in health systems, the impact of conflict on health-seeking behaviour and perceptions of childhood vaccinations. Data were analysed thematically using a conventional content analysis approach, allowing codes and themes to emerge inductively.

**Results:**

Health-seeking behaviour was shaped by a complex interplay of poverty, dysfunctional health infrastructure and profound mistrust of governmental institutions, all of which were exacerbated by prolonged exposure to violence. Patriarchal norms played a central role in decision-making processes, often limiting women’s autonomy when it came to accessing care. Vaccine hesitancy was influenced by misinformation, knowledge gaps and limited community engagement. However, caregivers with access to reliable information sources, such as community networks and local media, showed more positive attitudes towards immunisation.

**Conclusions:**

Efforts to improve maternal and child health in conflict-affected regions must prioritise trust-building, strengthening the health system in a culturally sensitive way and implementing targeted communication strategies. Leveraging community engagement and resilience is essential to reducing barriers to care and address vaccine hesitancy in fragile contexts.

## Introduction

Child health is a central priority in global health and a critical indicator of health equity, social justice, and the overall performance of health systems. Under-five mortality remains a key marker of population health and development, especially in low- and middle-income countries (LMICs). Despite significant global progress in child health, stark disparities persist [1].

In 2022, an estimated 4.9 million children under five died worldwide, nearly half of them in sub-Saharan Africa (SSA), where most deaths are due to preventable or treatable causes [2, 3]. These inequalities are most pronounced in settings affected by poverty, weak health system and prolonged conflict, which together constrain access to essential maternal and child health services, including routine immunisation. Nigeria has one of the highest under-five mortality rates globally, with recent estimates of 111 deaths per 1,000 live births [7], far exceeding the Sustainable Development Goal (SGD) target of 25 deaths per 1,000 live births by 2030 [4, 5]. National averages cover significant subnational disparities. In 2021, under-five mortality ranged from 52 deaths per 1,000 live births in the south-west to 253 per 1,000 in the north-west [6, 7]. In the northeast, where armed conflict has persisted for more than 15 years, child health indicators are among the worst in the country. One key driver of high child mortality rates in this region is limited access to healthcare services, particularly within communities affected by ongoing conflict.

For caregivers living under the constant threat of armed attacks, health-seeking decisions depend not only on perceived needs and service availability, but also on the pervasive instability caused by ongoing violence [7]. Health-seeking behaviour, understood as the actions individuals take to maintain, restore or improve health, results from a combination of social, cultural, psychological and structural determinants rather than individual choice alone [8]. In northeast Nigeria, where socio-political instability and violence are widespread, caregivers’ decisions are shaped by experiences of conflict, trust in institutions, distance to facilities, costs of treatment and cultural practices, all of which also influence attitudes towards preventive services such as vaccination [9–13].

The past years of insurgency have severely damaged the region’s health infrastructure. Boko Haram, which emerged in the early 2000s as a small religious movement, escalated into a large-scale armed insurgency after the uprising in Maiduguri and the killing of its leader Mohammed Yusuf [14]. The conflict has continued for more than 15 years and has since generated far-reaching humanitarian and health system consequences [15]. Even before the conflict escalated, the Northeast had the worst health indicators in Nigeria, with under-five mortality of 168.2 deaths per 1,000 live births in 2003 and immunization coverage below 2% in some states [16–18].

Conflict has further eroded the availability and quality of maternal and child health services. In the BAY states, more than 40% of health facilities have been damaged, destroyed, or rendered non-functional, reducing utilisation of services such as antenatal care (ANC) services and visits [15, 19, 20]. Excessive use of force against civilians by security forces has intensified an already pervasive climate of fear, creating additional barriers that discourage families from accessing essential care [7, 21].

### Exposure to armed conflict and vaccine hesitancy

Armed conflict has important indirect effects on child health, including disruptions to immunisation services and reduced uptake of maternal health care [19, 22]. Interruptions in vaccination campaigns can lead to resurgence and spread of preventable diseases and undermine trust in health authorities, thereby exacerbating vaccine hesitancy among populations exposed to violence [10, 23]. Concerns are particularly acute for preventive services such as immunisation, where fears about vaccine safety, perceived government neglect and socio-political rumours that heighten hesitancy.

Vaccine hesitancy is a prominent challenge in Nigeria [1, 13, 24]. The World Health Organization defines vaccine hesitancy as "delay in accepting or refusing vaccines despite the availability of immunisation services", emphasising that it is shaped by historical, cultural and political factors [25]. In northeast Nigeria, contemporary mistrust of government-led immunisation programmes is often traced to the 2003 polio vaccine boycott, when political and religious leaders in several northern states suspended vaccination campaigns after rumours that the vaccine was contaminated with anti-fertility agents and other harmful substances [26–29]. This nearly year-long boycott, rooted in longstanding distrust of federal authorities, geopolitical tensions and concerns about Western influence, not only halted vaccination activities during at the time but also fostered enduring scepticism toward routine immunisation [27, 30].

Evidence from Nigeria and other sub-Saharan African countries shows that vaccine hesitancy and incomplete vaccinations are driven by multiple, intersecting social and structural factors. A systematic review of reasons for non-vaccination in sub-Saharan African highlighted caregivers’ limited knowledge about vaccinations, time constraints, shortages of vaccines and health workers, and beliefs about vaccination as key contributors to incomplete immunisation [31]. Additional studies in Nigeria identified pressure from husbands or partners, as well as other cultural or religious norms, as further barriers to vaccinating children [32–34].

### Gender dynamics and decision-making

Gender relations play a central role in health-seeking behaviour and vaccination decisions in Nigeria’s predominantly patriarchal context. Women often have limited autonomy to make decisions regarding their own health and that of their children [26], as healthcare choices typically require approval from the male head of household [35]. Although mothers are the primary caregivers, male relatives frequently act as primary health decision-makers, sometimes despite limited knowledge of maternal and child care [26, 36, 37].

As a result, the education level, employment status, household wealth and awareness of the benefits of vaccination among male heads of household directly influence whether children receive preventive and curative services [38–42]. Studies indicate that women with greater decision-making autonomy have significantly greater odds of using ANC and childhood immunisation services compared to women with limited autonomy [43–45]. Understanding how gender norms intersect with conflict and structural barriers is therefore crucial to improving maternal and child health in this setting.

## Objectives

This study explores how health-seeking behaviour, exposure to conflict, trust in health systems and vaccine hesitancy intersect to shape caregivers’ decisions about accessing healthcare in northeast Nigeria. Drawing on qualitative data from focus group discussions with caregivers of children under five, the study seeks to develop an in-depth understanding of the barriers to and enablers of healthcare access in conflict-affected communities, and to inform culturally sensitive interventions tailored to their needs.

## Methods

### Setting and participants

This qualitative study was conducted in three conflict-affected states of northeast Nigeria (Borno, Yobe and Adamawa) in communities significantly affected by ongoing armed violence. The communities were purposively selected based on their documented exposure to Boko Haram-related violence and variation in conflict intensity across the three states: Borno, with the highest number of fatalities and conflict events; Yobe, with moderate but sustained insecurity; and Adamawa, which is comparatively less affected yet still experiences periodic attacks.

Data were collected in June 2024 through nine focus group discussions (FGDs) with caregivers of children under five years of age, conducted in local language (Hausa). Focus groups were chosen to encourage interaction and the exchange of ideas among participants, allowing for a deeper understanding of shared experiences and perspectives. Each FGD included eight participants and lasted approximately 60-90 minutes, in line with guidance recommending 6-10 participants per group to ensure diversity of perspectives and sufficient depth of discussion [46, 47].

Each FGD included eight participants and lasted approximately 60-90 minutes, in line with guidance recommending 6-10 participants per group to ensure diversity of perspectives and sufficient depth of discussion [46, 47] (Table 1). Inclusion criteria required participants to (i) be primary caregivers of at least one child under five and (ii) have primary responsibility for decisions about the child´s healthcare. The research team from Kano (co-author RJ), Yobe (co-authors AK, MJ), Borno (co-authors AA, MB) and Adamawa (co-authors MJ, AM) partnered with local community leaders who had established relationships in conflict-affected communities to identify potential participants. Identified individuals were approached face-to-face by culturally and linguistically matched research team members, who explained the purpose of the study, obtained informed consent and scheduled FGDs at convenient community locations.

**Table 1:** Sociodemographic characteristics of the participants

Participant ID	FGD ID	Gender	Age	Number of children	Occupation	District
001	B1	Female	33	9	Tailor	Borno
002	B1	Female	30	5	Home-based business	Borno
003	B1	Female	34	7	Tailor	Borno
004	B1	Female	43	9	Saleswoman	Borno
005	B1	Female	42	8	Saleswoman	Borno
006	B1	Female	39	4	Saleswoman	Borno
007	B1	Female	25	8	Saleswoman	Borno
008	B1	Female	30	2	Saleswoman	Borno
009	B2	Male	41	8	Director	Borno
010	B2	Male	38	6	Businessman	Borno
011	B2	Male	26	2	Civilian JTF	Borno
012	B2	Male	28	3	Undisclosed	Borno
013	B2	Male	24	4	Undisclosed	Borno
014	B2	Male	30	3	Civil Servant	Borno
015	B2	Male	32	8	Unclear	Borno
016	B2	Male	23	5	Teacher	Borno
017	B3	Female	28	4	None	Borno
018	B3	Female	48	10	None	Borno
019	B3	Female	31	3	Tailor	Borno
020	B3	Female	43	6	None	Borno
021	B3	Female	21	1	None	Borno
022	B3	Female	20	2	None	Borno
023	B3	Female	49	9	None	Borno
024	B3	Female	52	10	None	Borno
025	Y1	Female	24	3	Tailor	Yobe
026	Y1	Female	40	5	Businesswoman	Yobe
027	Y1	Female	25	2	Saleswoman	Yobe
028	Y1	Female	23	1	Saleswoman	Yobe
029	Y1	Female	24	1	Tailor	Yobe
030	Y1	Female	25	1	Tailor	Yobe
031	Y1	Female	25	3	Saleswoman	Yobe
032	Y1	Female	21	1	None	Yobe
033	Y2	Female	42	6	Teacher	Yobe
034	Y2	Female	25	3	None	Yobe
035	Y2	Female	23	2	Teacher	Yobe
036	Y2	Female	37	6	Undisclosed	Yobe
037	Y2	Female	39	3	Teacher	Yobe
038	Y2	Female	38	4	Teacher	Yobe
039	Y2	Female	28	3	Homebased Business	Yobe
040	Y2	Female	24	2	Homebased Business	Yobe
041	Y3	Male	35	7	Businessman	Yobe
042	Y3	Male	33	4	Businessman	Yobe
043	Y3	Male	38	5	Businessman	Yobe
044	Y3	Male	37	8	Farmer	Yobe
045	Y3	Male	45	17	Farmer	Yobe
046	Y3	Male	50	18	Farmer	Yobe
047	Y3	Male	40	11	Farmer	Yobe
048	Y3	Male	40	7	Traditional Bonesetter	Yobe
049	A1	Female	43	7	Farmer	Adamawa
050	A1	Female	33	6	Farmer	Adamawa
051	A1	Female	35	2	Farmer	Adamawa
052	A1	Female	37	5	Farmer	Adamawa
053	A1	Female	27	2	Civil Servant	Adamawa
054	A1	Female	20	2	Tailor	Adamawa
055	A1	Female	20	2	Farmer	Adamawa
056	A1	Female	25	2	Farmer	Adamawa
057	A2	Female	35	7	Saleswoman	Adamawa
058	A2	Female	50	3	Farmer	Adamawa
059	A2	Female	50	5	None	Adamawa
060	A2	Female	37	5	Saleswoman	Adamawa
061	A2	Female	44	3	Farmer	Adamawa
062	A2	Female	27	1	Saleswoman	Adamawa
063	A2	Female	46	3	Homebased Business	Adamawa
064	A2	Female	48	7	Homebased Business	Adamawa
065	A3	Male	24	1	Civil Servant	Adamawa
066	A3	Male	35	2	Farmer	Adamawa
067	A3	Male	28	2	Trader	Adamawa
068	A3	Male	61	11	Farmer	Adamawa
069	A3	Male	38	3	Trader	Adamawa
070	A3	Male	53	11	Farmer	Adamawa
071	A3	Male	33	3	Businessman	Adamawa
072	A3	Male	29	1	Farmer	Adamawa

The FGDs were gender-segregated, with separate discussions held for women and men to enhance cultural appropriateness and participant comfort. Moderators were matched by gender: female moderators facilitated women´s groups and male moderators facilitated men´s groups. All discussions were conducted in local language, Hausa, and followed a semi-structured interview guide covering experiences of conflict, health-seeking behaviour, trust in health systems and attitudes towards childhood vaccination.

### Data collection

The focus group discussions were facilitated by a team of trained Nigerian researchers residing in the three study states (co-authors RJ, AA, MB, AK, MJ and AM) – all experienced health professionals and researchers with extensive expertise in clinical practice, maternal and child health, public health research, and health service delivery in conflict-affected settings. Before data collection, all facilitators participated in an intensive one-day training workshop held in Kano in January 2024, led and supervised by authors (LA, MS). The workshop focused on qualitative research in conflict-affected and culturally sensitive settings and covered building rapport and trust with participants; trauma-informed interviewing; recognising and managing distress; maintaining confidentiality and safe data handling; preserving neutrality and reflexive awareness; and ethical considerations in insecure environments.

During the training, the full FGD guide was jointly reviewed and refined with the local research team to ensure clarity, contextual relevance and appropriateness of all questions. As elaborated in the Reflexivity Statement, facilitators’ linguistic fluency, cultural competence and embeddedness in the local communities strengthened the quality, safety and sensitivity of data collection.

To safeguard participants’ wellbeing given the potentially distressing nature of topics discussed, facilitators monitored emotional comfort throughout each FGD. Participants were informed that they could decline to answer any question, pause, or withdraw at any time without negative consequences. At the end of each session, participants received information on available local psychological and health support services.

All FGDs were audio-recorded for transcription with participants´ permission. A note-taker (co-authors MJ, AM, AK, AA, and MB) attended each session to document non-verbal communication, group dynamics and contextual observations in field notes.

### Data analysis

Audio recordings were transcribed verbatim in Hausa and translated into English. Bilingual researchers checked the transcripts for accuracy against the original recordings. Data were analysed using a conventional, content-driven thematic analysis approach, following the steps outlined by Braun and Clarke [48].

Two researchers (co-authors LA, and PS) independently read and re-read the transcripts and field notes to familiarise themselves with the data and identify initial codes. Key phrases and statements were systematically coded to capture meaningful segments related to the research questions. Codes were then grouped into broader categories, from which themes and sub-themes were developed and iteratively refined to ensure they captured the underlying patterns in the data. Discrepancies in coding or theme development were resolved through discussion, and final themes were agreed by consensus among co-authors [1–3].

Data were managed and organised using Nvivo qualitative data analysis software (Release 1.7, QSR International). Consistent with a reflexive thematic analysis perspective, saturation was conceptualised as “thematic sufficiency”: as the analysis progressed, no substantially new concept emerged in the final FGDs, indicating that the dataset provided sufficient depth and richness to address the research questions [49].

### Ethics approval and consent to participate

Ethical approval was obtained from the Ethical Committee from the University of Konstanz (IRB statement 13/2024) and the National Health Research Ethics Committee of Nigeria NHREC/01/01/2007-14/01/2024). All procedures complied with national and institutional guidelines for research involving human participants.

All participants provided written informed consent before data collection. For participants with limited literacy, the consent form was read aloud in their preferred language and consent was documented with a thumbprint. Participants were informed of the voluntary nature of participation, their right to withdraw at any time without consequences and the measures in place to protect their confidentiality.

### Data protection, confidentiality and reporting standards

All data were anonymised immediately after transcription. Personal identifiers - including names, specific locations and any details that could reveal participant identity - were removed or replaced with coded labels. Audio recordings and anonymised transcripts were stored securely on encrypted, password-protected institutional servers accessible only to authorised members of the research team. Quotations used in the manuscript were checked to ensure that they did not contain potentially identifying contextual information.

The study adhered to the Consolidated Criteria for Reporting Qualitative Research (COREQ) 32-item checklist for interviews and focus groups, which provides guidance on transparent reporting of research team characteristics, study methods, context, analysis and findings [50]. This included documenting details of the research team, data collection and analysis processes, and providing a clear audit trail of coding and theme development.

### Consent for publication

Because the manuscript includes individual-level qualitative data (e.g. direct quotations), explicit consent for publication was obtained from all participants in addition to consent for participation.

Participants were first approached by trained local researchers, who explained the purpose of the study, what participation involved, and their rights, including confidentiality and voluntary withdrawal. Individuals who agreed to take part were invited to attend a scheduled FGD. On the day of data collection, the study information was reviewed again and questions were addressed before obtaining written informed consent from all participants.

To protect participant wellbeing during discussions of potentially distressing experiences, facilitators were trained in trauma-informed interviewing and monitored participants´ emotional comfort throughout the FGDs. Participants were informed that they could decline to answer any question, take a break or withdraw at any time without negative consequences. At the end of each session, participants were provided with information about local psychosocial and health support services.

### Reflexivity statement

Reflexivity was integral to all stages of the research process, from study design to data interpretation. The research team included Nigerian and international researchers with expertise in global health and qualitative research methods. The Nigerian team brought in-depth knowledge of the language, cultural norms and social dynamics of northeast Nigeria. Throughout data collection, Nigerian researchers (co-authors RJ, AA, MB, AK, MJ and AM) sought to minimise power differentials by ensuring that FGD facilitators were culturally and linguistically matched to participants.

Regular team meetings were held during data collection and analysis to reflect critically on field experiences, emerging themes and potential biases. These discussions, supported by field notes and analytic memos, helped the team remain aware of how their positionality and assumptions could shape data interpretation and ensured a more nuanced and contextually grounded analysis.

## Results

A total of nine FGDs were conducted in June 2024 across three states in northeast Nigeria: three FGDs each in Borno (n=24), Yobe (n=23), and Adamawa (n=24). Six FGDs were composed exclusively of women (n=48), while three FGDs included only men (n=24), resulting in a total of 72 participants (see Table 1). Participants reported having between 1 and 18 children, accounting for 355 children in total. Although most participants indicated that they had some form of occupation, women were more likely than men to report having no formal employment or means of livelihood. Participants ranged in age from 20 to 61 years, with an average age of 34 years.

**Fig 1.**
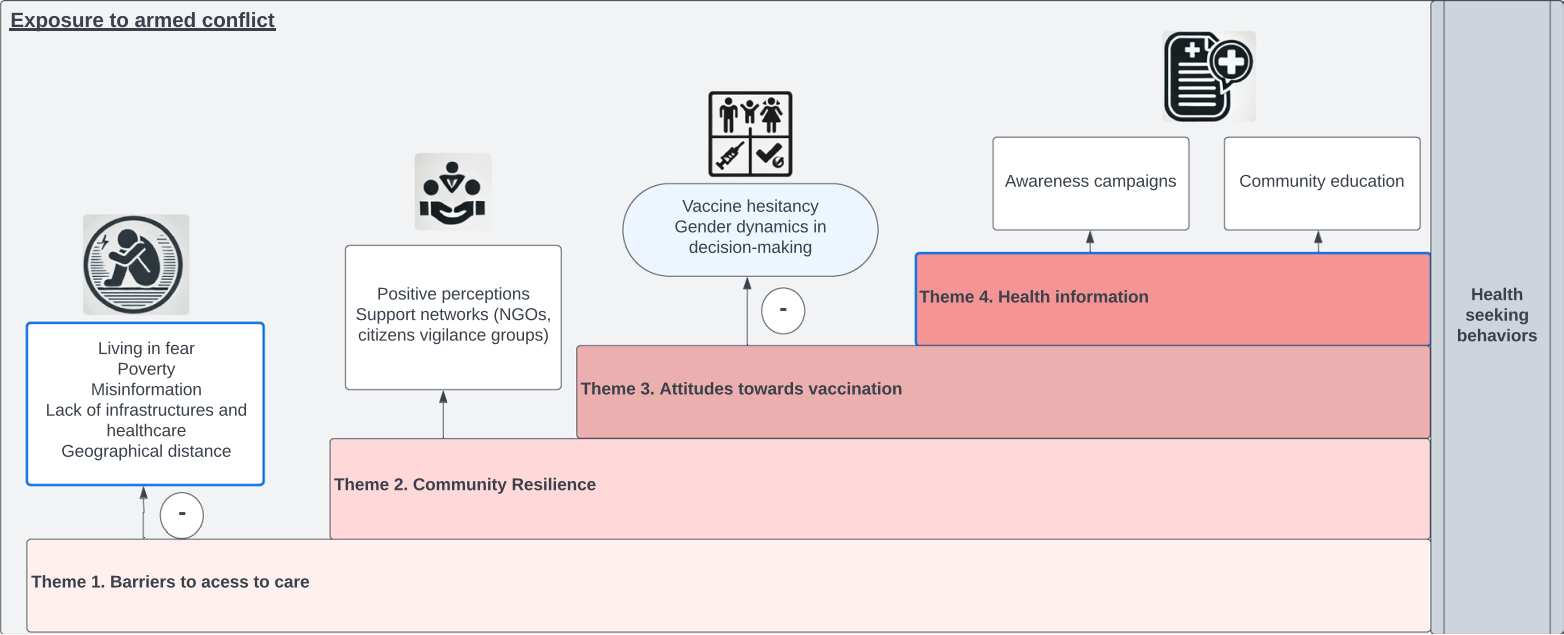
Main themes of health seeking behaviour of caregivers with children under 5 years old

Analysis of the nine FGDs with caregivers revealed a complex interplay between health-seeking behaviours, trust in health systems and the effects of ongoing violence. Participants described substantial barriers to accessing health care, community-driven strategies developed to overcome these challenges, and varied attitudes towards vaccination. The role of health information - how it is shared, interpreted and acted upon – also emerged as a critical factor shaping decisions. The analysis generated four interrelated themes (Figure 1) that together illustrate how caregivers navigate health-seeking in the context of protracted insecurity, structural constraints and shifting community dynamics. Each theme comprises several subthemes that capture distinct yet interconnected aspects of caregivers’ experiences. **Theme 1** - Barriers to accessing healthcare, describes the multi-layered obstacles that restrict access to services. **Theme 2** - Community resilience and adaptation, highlights the adaptive strategies and forms of resilience that emerge in response. **Theme 3 -** Attitudes towards vaccination, explores caregivers’ attitudes and decision-making around childhood vaccination within these constraints. **Theme 4** - Health information, focuses on how the information circulates and ways in which it influences behaviour.

Taken together, these themes present a coherent overview of how conflict, trust, gender dynamics and local support systems intersect to shape health-seeking behaviour in northeast Nigeria. Figure 1 reflects the order and relative size of the thematic blocks and their analytic prominence within the dataset, with Theme 1 appearing largest because it contained the greatest number of coded segments and participant references. This representation follows Braun and Clarke’s guidance that themes should be organised according to their centrality and extensiveness within the data rather than implying a temporal or causal sequence.

**Table 2:** Description of themes

Themes	Description
**1.** **Navigating barriers to accessing healthcare**	This theme describes main barriers of access to care. Caregivers consistently identified multiple barriers to accessing health services, exacerbated by the ongoing conflict. Fear of violence and insecurity often obstructed caregivers from visiting health facilities, further complicated by the destruction of infrastructure and the absence of health workers. Economic challenges, including widespread poverty and the cost of transport and treatment, were significant barriers. In addition, long distances to health facilities posed logistical and safety concerns, especially in emergencies. Together, these barriers limited caregivers’ ability to seek timely and appropriate care.
*1.1 Living in fear and insecurity*	
*1.2 Economic constraints*	
*1.3 Infrastructural deficits*	
*1.4 Geographical distance*	
**2.** **Resilience and community enabling strategies**	This theme describes collective mechanisms of resilience during conflict times. In the midst of the inherent conflict-related challenges, caregivers showed remarkable resilience through community-based strategies. Participants described how vigilante groups provided security and facilitated access to health care by escorting patients. Communities pooled resources for transportation and medicines, while local NGOs filled critical gaps left by the government, providing essential health services and support. Where formal healthcare was inaccessible, traditional herbalists were used as alternative. These enabling measures underscored the resourcefulness of communities in overcoming systemic failures in health care.
*2.1 Experiences of violence*	
*2.1 Support networks*	
*2.3 NGO involvement*	
*2.4 Integration of traditional practices*	
**3. Attitudes towards vaccination**	This theme describes attitudes regarding vaccination. Caregivers were generally positive about it, recognising its importance in preventing disease and protecting children’s health. However, significant barriers remained, including disruption of immunisation services due to conflict, and vaccine hesitancy due to fear of adverse effects. Gender dynamics influenced decision-making, with male household members often having the authority to agree or refuse vaccination. Community-led awareness campaigns played a key role in addressing these concerns, increasing acceptance of the vaccine and improving uptake rates.
*3.1 Positive perceptions*	
*3.2 Vaccine hesitancy*	
*3.3 Gender dynamics in decision-making*	
***4. Health information landscape***	This theme addresses health information. Participants highlighted the effectiveness of awareness-raising campaigns, particularly those involving trusted community leaders or broadcast on radio, in increasing knowledge about maternal and child health. These efforts helped to counter misinformation, particularly around immunization, but gaps in access to reliable information remained. Participants stressed the importance of sustained, context-specific health education initiatives to further improve health decision-making and outcomes in their communities.
*4.1. Role of awareness campaigns*	
*4.2 Community education*	

**Theme 1: Navigating barriers to accessing healthcare**

This theme describes the major obstacles caregivers face when seeking healthcare in conflict-affected areas. Insecurity, financial hardship, damage infrastructure, and long distances to facilities combine to make both routine and emergency care difficult to access. Across all subthemes, women reported greater vulnerability, reduced mobility, and heavier caregiving burdens, illustrating the gendered nature of these barriers (see Table 3 for further quotations).

**Table 3:** Barriers to healthcare (Theme 1)

1 Navigating barriers to accessing healthcare
**1.1 Living in fear and insecurity**
[1.1a] “In this situation, the armed conflict, banditry and thieves really affected us seriously and it made it very difficult for us to access the healthcare services, because of this situation, even if you are sick in the night no one or not even an Okada (motorcycle) will agree to take you to the hospital because of fear and lack of trust on who is picking so it really affected our healthcare access in this community.” FGDB2, male, P10. [1.1b]B “There was a time we were at home around 7.00pm we heard gunshots and were told to run away but I could not run away I stayed on top of a hill outside the community. That time I was nine months pregnant, and because of that panic I delivered in the bush inside a trench.” FGDB3, female, P24.
**1.2 Economic constraints**
[1.2a] “When the insurgency started in our area, I was on the farm, I ran away and we didn’t go back home. At that time, we didn’t have money, my husband only had N300 naira in his pocket so we managed to go to his village where he left me under the care of his parents and went to Lagos. So, after he came back from Lagos we returned back to our village and after some time, these insurgents attacked us again and that´s when my husband was killed by the Boko Haram.” FGD**B3**, female, P17. [1.2b] “We lived in difficulties during the violent conflict and to access healthcare was very difficult because both my husband and my parents had fled. Yes, our health seeking behaviour has changed because we have not been able to access free healthcare services, we are even afraid to go out and take ourselves and our children to the hospital.” FGD**A1**, female, P56. [1.2c] “One of the barriers is poverty that stop caregivers from taking their children for vaccination; lack of involvement of community leaders is another factor; distance to the hospital is also another reason; and lack of transport can be a factor. Lack of provision of free drugs is another reason.” FGD**A2**, female, P58. [1.2d] “Poverty and financial constraints are the main barriers that prevent caregivers from taking their children for vaccination.” FGD**A2**, female, P59. [1.2e] “Some would like to visit hospital but lack of money prevents them from accessing care.” FGD**B1**, female, P3.
**1.3 Infrastructural deficits**
[1.3a] “There was a time when my child had an accident and was taken to the health facility but there were not enough resources to treat him. Then we were referred to secondary and then to tertiary institutions to treat him.” FGD**B1**, female, P3. [1.3b] “At that time, we went back there was no access to healthcare services because the health facilities were not operating and at the same time there was no drinking water, and this seriously affected our healthcare services. There was no food, and everything was in shambles there was no access to portable water, electricity and no good healthcare service.” FGD**B3**, female, P21.
**1.4 Geographical barriers**
[1.4a] “Yes, at that time there was no means of transport to take your child or pregnant woman to the hospital. We really lived in a difficult situation because my husband had run away to Enugu, and I have stopped hearing from him since the armed conflict began. I am now pregnant, and my expected date of delivery is this month of May (2024), I don’t know what to do, I am living in confusion. But at times my mother-in-law is assisting me with the little she gets.” FGD**B1**, female, P5. [1.4b] “Yes, for those that live in villages under this community it is not easy for them to transport themselves to the health facility because their houses are very far away from the hospital is one of the barriers they are facing. ” FGD**B1**, female, P3. [1.4c] “Yes, our behaviour has changed due to the fear of Boko Haram and also there was problem of transportation because of distance and also some other people live in the hard-to-reach areas. That is why our seeking behaviour has changed and some could not access care in the hospital.” FGD**Y3**, male, P48.

### Living in fear and insecurity

Participants described pervasive fear and insecurity resulting from ongoing violence, which profoundly restricted their ability to seek healthcare. The risk of being attacked, kidnapped or mistaken for insurgents created a constant state of alertness, making travel to health facilities dangerous. For many caregivers, fear extended beyond personal safety to concerns for their children when left at home:

“*We were afraid of being attacked on our way to the hospital and at the same time we were afraid that our children could be kidnapped by bandits in our absence.*” FGD1, Female, P8

Lack of transportation at night intensified these risks[1.1a]. In extreme cases, insecurity disrupted maternal healthcare entirely: one mother recounted being forced to deliver her baby in the bush while fleeing from an armed attack [1.1b].

*Gender analysis:* Women reported substantially higher levels of vulnerability, insecurity and trauma [1.1b, 1.2a, 1.4a, 2.1a-d]. Their narratives frequently described giving birth during violent events, losing husbands (often primary income earners), caring for children alone and coping with chronic fear, displacement, and economic hardship. Women’s mobility was further constrained when husbands fled, were killed, or were abducted. Although men acknowledged the general climate of insecurity [1.1a], women provided more direct and acute accounts of risk, especially during pregnancy, childbirth and caregiving.

### Economic constraints

Economic hardship emerged as another major barrier to healthcare access. Conflict-related loss of livelihoods, displacement, destructions of homes and farmland, and reduced income intensified pre-existing poverty. Caregivers explained that these factors directly affected their ability to afford transportation, medical fees or medication, thereby preventing both urgent and routine care, including vaccinations. One caregiver described how recurrent attacks and the death of her husband left her household unable to meet basic needs [1.2a]. Another participant summarised the situation more broadly:

“*The armed conflict really affected us seriously… We live in poverty because they have burnt everything, we are left with nothing. Even to buy medicine we can’t afford it*.” FGDA1, Female, P55

Participants also noted that the absence of free medication and financial support further limited their ability to seek care. Many emphasised that the willingness to use services - including vaccinations - was present, but financial constraints made access impossible. Economic hardship also disrupted routine health-seeking behaviour [1.2b-e]. 

**Gender analysis:** Women consistently reported the greatest financial strain [quotes 1.2a-e]. Many became sole caregivers following the death, disappearance or displacement of their husbands [1.2a]. Women described being unable to travel to health facilities when husbands had fled or were absent, compounding their economic and caregiving burdens [1.2b, 1.2e]. Financial constraints – particularly lack of money for transport, medication, food and basic needs - were a recurring challenge, regularly preventing them from seeking timely care for themselves or their children [1.2c-d]. In contrast, men rarely described economic hardship with the same personal intensity. Overall, women disproportionately suffer the financial consequences of disrupted livelihoods and single parenthood following male absence or death.

### Infrastructural deficits

Participants described severe conflict-related damage to health infrastructure, including destruction of facilities, migration of health workers and shortages of essential supplies. These deficits significantly impeded timely and appropriate care, particularly in emergencies:

*“Another issue was the lack of enough healthcare providers at the hospital, both during the day and at night; most of them have either migrated or fled to other places for their safety.* “ FGDB1, Female, P1

Caregivers often faced complex referral pathways without resolution (Table 3, 1.3a). Lack of clean water, electricity, and functioning facilities further compromised service availability (Table 3, 1.3b). Some participants recounted tragic outcomes, including child deaths from malaria and typhoid due to delayed or absent care:

*“… child was affected by malaria and typhoid but due to lack of care from the hospital and the inability of healthcare, he died.” FGDB3, Female, P19*

### Geographical barriers

Distance to health facilities was another major barrier. One participant described being pregnant, displaced and without access to transport or family support [1.4a]. For those living in remote villages, long distances alone prevented care-seeking [1.4b]. Poor road conditions, especially during the rainy season, further limited access. The combination of fear of travelling and geographic isolation discouraged caregivers from seeking care [1.4c].

**Theme 2: Resilience and community enabling strategies**

This theme explores how caregivers and their communities cope with the challenges of living in conflict-affected settings. Despite exposure to violence, communities mobilised support networks, including vigilante groups, youth escorts and informal transport system, to enhance safety and maintain access to care. NGOs also played a crucial role in filling service gaps, while some caregivers relied on traditional practices when formal services were unavailable. Gender differences were evident, with men emphasising roles in protection and mobility, while women highlighted emotional strain and the practical demands of daily survival (see Table 4 for further citations).

**Table 4:** Resilience and community

2. Resilience and community enabling strategies
**2.1 Experiences of violence**
[2.1a] “Exposure to violence has affected our well-being in the sense that after my husband was killed by the Boko Haram insurgents, I was left to take care of my children, their schools fees, shelter, health and any other well- being. This is very difficult for me and I encountered serious challenges being a woman without a husband with some kids under my care. Yes, there are few people in the community that support me, but not 100%.” FGD**B1**, female, P8. [2.1b] “There were killings of innocent people within the community after we returned around April this year (2024). I witnessed and saw them lying on the ground dead. About 20 people were killed (both males and females, including young children) within or in some parts of our community after we have returned. As a result of that tragedy, accessing healthcare services become difficult because of fear and anxiety.” FGD**B3**, female, P17. [2.1c] “Yes, I was exposed to the violent conflict of Boko Haram, my husband ran away and left us with our children, and some were even shot, and my husband was kidnapped and stayed with them for over one year, he was later released.” FGD**Y1**, female, P26. [2.1d] “When I was in Gwoza, I was exposed to Boko Haram insurgency where my father was killed by the insurgents and my husband was also killed. It was an extremely terrifying period.” FGD**Y1**, female, P27.
**2.2 Support networks**
[2.2a] “Yes, there is this committee of hunters and vigilantes that patrols the area in the night so that those bandits do not attack our community. They also have the support of government security agencies in protecting the community.” FGD**B2**, male, P14. [2.2b] “We used the “No Boko Group” that assists in protecting the community and in fighting against the armed bandits. We also pay them some allowance at the end of every month and salary/allowance come through the emir’s palace, stakeholders, and philanthropists and this will motivate them to protect the community.” FGD**B2**, male, P15. [2.2c] “We built a gate at the entrance of the community so that any person that is coming into the community will be monitored and security is manned at the gate entrance and this has improved the security situation of our community.” FGD**B2**, male, P16. [2.2c] “There is a group of youths that used to escort us (to the hospital) also in the night if there is a person that is seriously sick or when a woman is in labour.” FGD**A3**, male, P68.
**2.3 NGO involvement**
[2.3a] “At that time my daughter was sick and we were unable to access care in the hospital, but we have the numbers of the NGO phone and when the illness became complicated, we were asked to go to Maiduguri for further care. We went to one NGOs hospital in Maiduguri, and we received good medical care at that time.” FGD**Y1**, female, P28. [2.3b] “I think these NGOs are better than the government but also the government is trying.” FGD**Y1**, female, P25. [2.3c] “But on our return to our community, accessing healthcare became very difficult. There were no adequate drugs in the health facility we attended, no adequate healthcare personnel. We don’t even have money to buy the medicines, let alone to transport ourselves to the hospital. This insecurity issues and violent conflict have affected us seriously. At that time my child was affected by malaria and typhoid, and due to lack of care from the hospital and the inability of professional health personnel, he died.” FGD**B3**, female, P19.
**2.4 Integration of traditional practices**
[2.4a] “What made us change our health-seeking behaviour was that if you went to the hospital, you would not see any health professional on duty. So, we go back to the herbalist woman to check our pregnancy. “ FGD**B3**, female, P20.

### 2.1 Experiences of violence

Caregivers shared profound accounts of violence, loss and trauma. Many described how the insurgency disrupted all aspects of life, including their ability to care for their families or seek medical treatment. Losing a spouse placed an immense emotional and economic strain on caregivers [2.1a]:

“*I felt bad because I have orphans that I am now taking care of … My husband… was shot… and it was a bad moment for me.*” FGDB1, Female, P4

Participants also described witnessing mass killings, displacement, abduction, and family separation, all of which generated ongoing fear and hindered healthcare access (Table 3, 2.1b-d).

### Support networks

Despite the challenges posed by violence, communities mobilised to support one another. Vigilante groups, youth escort teams, and community patrols were formed to enhance security and facilitate access to care: (Table 3, 2.2a):

“*We formed a group of vigilantes that control the security situation in the night… when the bandits come… so that we can have peace in the community… Many neighbouring communities did the same*.” FGDB2, Male, P12

The "No Boko Group" was described as a key community-led security initiative, widely praised for improving safety, organising patrols, and implementing a sustainable funding model through monthly community contributions (Table 3, 2.2b). Additional security measures, such as building a monitored community gate, reinforced safety (Table 3, 2.2c). Several caregivers noted that the with the “No Boko Group” and the community security structures, such as gate checkpoints and youth escort teams, there was a greater sense of safety, particularly at night. Youth groups escorted sick community members and pregnant women during emergencies (Table 3, 2.2c).

These illustrate how informal community security structures became critical sources of resilience. By organising local initiatives and leveraging collective resources, these communities were able to address some of the barriers to healthcare access and create a sense of security.

### NGO involvement

Non-governmental organisations (NGOs) played an essential role in bridging gaps created by weakened health systems. Participants described relying on NGO-supported hospitals for specialised care (Table 3, 2.3a) or during displacement:

“*… we went to one NGO hospital in Maiduguri, and received good medical care* “FGDB1, Female, P04

NGOs were frequently perceived as more reliable and responsive than government services (Table 3, 2.3b). Their presence was particularly vital during emergencies when local facilities were understaffed or overwhelmed.

### Integration of traditional practices

Due to the absence or poor functionality of formal health services, caregivers often turned to herbalists and traditional medicine for urgent care. Participants explained that the lack of available health workers sometimes forced to seek alternative methods, such as consulting herbalists for pregnancy monitoring (Table 3, 3.4a).

For many, access to formal health services symbolised safety and normalcy – conditions disrupted by conflict.

**Gender analysis:** Clear gendered differences emerged in how men and women described their roles and responsibilities within the conflict context. Men predominantly framed their contributions in terms of protection and logistical support, emphasising their involvement in community defence groups and vigilance activities [2.2a-c], escorting residents to health facilities during emergencies [2.2c], and providing transport for their wives to access maternal or child healthcare [3.3a]. In contrast, women focused on the emotional and practical burdens of daily survival. Their narratives centred on caring for children alone in the absence of husbands [1.2a; 2.1a-d], coping with fear and insecurity [1.1b; 1.2b], and navigating disrupted maternal and child health routines, such as pregnancy, childbirth and seeking healthcare under dangerous conditions [1.1b; 1.4a]. These contrasting perspectives indicate that while men primarily positioned themselves as protectors and facilitators of mobility, women´s experiences were more closely tied to caregiving work, emotional strain and the direct impacts of conflict on maternal and child health.

**Theme 3: Attitudes towards vaccination**

This theme examines caregivers’ perceptions of childhood vaccination, revealing a mix of strong appreciation for its benefits and persistent concerns shaped by misinformation, fear of side effects and mistrust of government programmes. While awareness campaigns and community leaders helped improve acceptance, rumours and historical distrust continued to fuel hesitancy. Gender dynamics played a central role: men often acted as key decision-makers influencing vaccination uptake, while women described navigating family pressures, misinformation and limited autonomy in seeking services (see Table 5 for further citations).

**Table 5:** Attitudes towards vaccination

3. Attitudes towards vaccination (Theme 3)
**3.1 Positive perceptions**
[3.1a] “The majority of people now understand the importance and benefits of vaccination for their children. In the past people did not believe in vaccines due to ignorance, illiteracy, and lack of awareness about the benefits of vaccines. In the past, people were hiding their children from vaccination. But when the polio vaccine started having a positive effect on our children’s suffering from polio, other caregivers came to understand about the importance of vaccines, and they started allowing their children to be vaccinated. People thought that the government was trying to reduce the number of the people in a society, but due to campaigns they now understand that this is not true. As for the risk, I don’t think vaccination has any risk to the lives of our children. The only risk I can say is that if the doctor or healthcare worker is not an expert in vaccination, he might do it in the wrong way or place and this will make a child’s leg to swell”. FGD**B2**, male, P16. [3.1b] “I am a teacher and at times *vaccinators* used to visit our schools to vaccinate our children against polio and other related diseases. Recently, as I recall, there was an intervention campaign on vaccines for 9 and 14 year-olds and no one agreed to be vaccinated. And the headmaster did not allow them to vaccinate the children because he was having challenges with the caregiver. The vaccine is to prevent cervical cancer, but some agreed to be vaccinated and others refused. The reason for their refusal is also a result of rumours about the side effects of the vaccine.” FGD**B1**, female, P4.
**3.2 Misinformation as a barrier**
[3.2a] “Usually what makes them not to vaccinate their children is the adverse effect of some vaccines like DPT, which makes the child cry or fall sick and have a swollen leg. This often stops them from coming back to take or complete the vaccination.” FGDB3, female, P19. [3.2b] “Some are suspecting that the vaccine is used for family planning so that in the future their children will not bear children.” FGD**Y1**, female, P32. [3.2c] “There is a lack of awareness about the importance of the vaccine. This is the reason why some do not bring their children for vaccination. It is ignorance and illiteracy.” FGD**Y2**, female, P34. [3.2d] “The lack of free nutritional supplements for the children is what discourages caregivers from taking their children for vaccination. Because in the past children were given incentives after each dose of vaccine.” FGD**A2**, female, P58. [3.2e] “Yes, there is believe that it is plot to decrease the population of our people in the community. That is why some caregivers don’t take their children for vaccination.” FGD**A2**, female, P57. [3.2f] “The reason why some people do not like to vaccinate their children as I have said is illiteracy, ignorance and lack of awareness about the importance and benefits of vaccination. Everybody knows prevention is better than cure, so for those who do not allow their children to be vaccinated, it is mainly due to illiteracy.” FGD**B2**, male, P15. [3.2g] “But in my own view, this issue of exclusive breastfeeding is the one that has made our children to misbehave and do not respect the elders because they were not given water when they were born as the western people directed. That is the reason why now most of the youths whose mothers practiced exclusive breastfeeding do not respect their elders.” FGD**B1**, female, P4. [3.2h] “Some of their husbands do not allow them to vaccinate their children and they think that it is going to make their children sterile in the future.” FGD**Y1**, female, P28.
**3.3 Gender dynamics in decision-making**
[3.3a] “Yes, the decision to seek health might change because of lack of transport to take your wife to the hospital for maternal healthcare; but for me, nothing has changed because I must find a way of taking my family to the hospital to access care services, be it maternal or any other healthcare services that we need. Yes, I am afraid but I had no choice but to seek healthcare service(s) at that particular time.” FGD**B2**, male, P16. [3.3b] “In fact, even the husbands that do not allow their wives to take their children for immunisation now all agree and even encourage it. There is a lot of awareness.” FGD**Y2**, female, P34. [3.3c] “One of the reasons is that my husband doesn’t like it because of the side effects. But my in-laws make sure that our children are vaccinated.” FGD**A2**, female, P63. [3.3d] “My in-laws do not like our child to be vaccinated, but I refused their advice and took my child for vaccination.” FGD**A2**, female, P60. [3.3e] “There is need for increased awareness in the community about the importance and benefits of vaccines, and the majority of caregivers who have doubts about vaccines are fathers or husbands. In my view, their concern is related to tradition and religious beliefs that prevent them from allowing their children to be vaccinated. But there is a commitment from the chief Imams of the area to include the issue of vaccination in their sermons, telling the public about the importance of the vaccine for people.” FGD**B1**, female, P6.

### 3.1 Positive perceptions

Participants commonly recognised the benefits of vaccination for preventing childhood diseases. Awareness campaigns, religious leaders, and community gatherings were credited with improving understanding and uptake:

“*The health of our children has improved because most of those diseases have been eradicated because of vaccination… This was a result of raising awareness in the community either in the mosques, churches or social gatherings*.” FGDB2, Male, P13

Participants also referred to historical barriers to vaccination, such as ignorance and misinformation, and how these had been mitigated through education efforts (Table 5, 3.1a). Notably, some participants also used the term *vaccinators* to describe door-to-door health workers (Table 5, 3.1b).

### 3.2 Misinformation as a barrier

Despite positive perceptions, misinformation fuelled hesitancy. Rumours about side effects, infertility and population-control motives discouraged some caregivers:

“*…they believe the rumours about the side effects and efficacy of vaccines. That is why they do not allow their children to be vaccinated*.” FGDB1, Female, P3

Side effects such as swelling or fever after vaccination sometimes prevented caregivers from completing vaccination schedules (Table 5, 3.2a). Myths linking vaccines to family planning or infertility were commonly cited (Table 5, 3.2b). Illiteracy and lack of awareness further contributed to hesitancy (Table 5, 3.2c).

The withdrawal of incentives (e.g., nutritional supplements previously given during immunisation campaigns) further discouraged participation (Table 5, 3.2d, 3.2e, 3.2f, 3.2g).

###  Gender dynamics

Traditional gender roles meant that women often required permission from husbands or in-laws to access health services, including vaccination. Male participants described varying degrees of support or restriction:

“*For me, I will not allow my wife to attend ANC services during these times of conflict… I prefer to visit the hospital during daylight.”* FGDB2, Male, P16

Some women described resistance from husbands or inlaws, yet many insisted on vaccinating their children despite opposition (Table 5, 3.3c-e).

Gender analysis: Men frequently positioned themselves as the primary decision-makers regarding when and whether their wives or children could access care [3.3a]. Concerns about safety, tradition, or vaccine side effects sometimes led them to restrict vaccination [3.2b; 3.2h].

Women’s narratives centred on balancing caregiving responsibilities with managing fear, navigating family pressures, and ensuring child health under dangerous conditions [3.3d; 3.3e]. Even when men expressed supportive attitudes, their statements reinforced gendered gatekeeping roles.

**Theme 4: Health information landscape**

This theme highlights the central role of health information in shaping caregivers’ decisions. Awareness campaigns led by trusted community and religious leaders, along with radio messaging, helped rebuild confidence in maternal and child health services. Community-based education – such as door-to-door outreach and visual materials – supported timely care-seeking and countered misinformation (see Table 6 for further quotes).

**Table 6:** Health Information

4. Health information landscape (Theme 4)
**4.1 Role of awareness campaigns**
[4.1a] “Today, the majority of the women deliver their babies in the hospital because they are aware and understand the importance and benefits of delivery in hospital, and also because they fear complications during labour.” FGD**Y3**, male, P44. [4.1b] “It is the awareness on the importance of delivery in the hospital that leads most of the women to deliver in hospital. Because hospital deliveries prevent the risk of complicated issues.” FGD**Y3**, male, P42. [4.1c] “We thank God because of this awareness, the majority of our women now deliver in the hospital; they now understand that hospital delivery is safer. They also know the importance of ANC and other maternal services like postnatal appointments.” FGD**Y3**, male, P46.
**4.2 Community education**
[4.2a] We are very happy to see or hear of any health-related information like the one that they used to go around the community educating the people about the importance of healthcare and on how to take care of yourself and your children. Also, the use of posters showing the signs or symptoms of diseases educates and creates awareness that if they see such symptoms of illness they should rush to the hospital for care. FGD**Y3**, male, P43. [4.2b] “There is a community programme that promotes and supports childhood vaccination. They used to go from house to house to educate community members about the importance and benefits of vaccinations. This campaign involved the team of healthcare providers and the community leaders, that is, the ward heads, religious leaders and other philanthropists that reside in that community.” FGD**B1**, female, P4. [4.2c] “These posters and leaflets encourage us and make us happy to see or look at health related information that will improve our lives and the lives of our children, because it will educate us to know the importance of vaccination, as well as other child and maternal health issues.” FGD**A2**, female, P60. [4.2d] “Yes, it helps us to know the symptoms of diseases so that if you come across any, you can immediately take your child to the nearest hospital for treatment; also, the information helps us to bring our children for vaccination because it is very beneficial.” FGD**A2**, female, P61.

### Role of awareness campaigns

Participants emphasised the positive influence of health promotion activities, particularly those delivered through trusted religious leaders, community figures or radio broadcasts. These campaigns increased acceptance of hospital-based births, antenatal and postnatal care, and other maternal health services (Table 6, 4.1a-c). Awareness campaigns also helped rebuild trust in formal health services following years of conflict-related disruption [Table 6, 4.1c].

### 4.2 Community education

Caregivers valued community-based education strategies, including door-to-door visits, small group sensitisation sessions, and the use of visual materials such as posters and pamphlets. One participant highlighted the usefulness of posters illustrating disease symptoms and appropriate actions [Table 6, 4.2a], and how such visual materials were especially important in low-literacy settings [Table 6, 4.2c, 4.2d]. House-to-house campaigns involving health workers, traditional leaders, and local philanthropists were described as highly effective in promoting vaccination and building trust [Table 6, 4.2b].

*Gender analysis:* While both men and women reported exposure to misinformation, women described its interpersonal and emotional consequences more vividly - fears about infertility [3.2b; 3.2h] or pressure from mothers-in-law [3.2e]. These accounts illustrate how misinformation interacts with gendered family hierarchies and social expectations, shaping women´s autonomy in health decision-making. Men tended to discuss misinformation in more general terms, focusing less on family dynamics and more in community-level mistrust [3.2f].

## Discussion

This study provides a comprehensive examination of health-seeking behaviour, trust in health systems and vaccine hesitancy among caregivers of children under five in conflict-affected regions of northeast Nigeria. The findings reveal how insecurity, poverty, infrastructural collapse and embedded gender norms interact to shape caregivers’ decisions and behaviours. Notably, the gender-sensitive analysis demonstrates that these dynamics are not experienced uniformly: gender acts as a critical axis of vulnerability, responsibility and decision-making.

Pervasive insecurity is characterised by recurrent attacks, kidnapping, and targeted violence and created a climate of sustained fear that severely limited caregivers’ mobility and access to healthcare. Consistent with evidence from other conflict-affected settings [19], participants frequently reported delaying or avoiding healthcare, particularly maternal and child health services, due to the risks associated with travelling. For women, these risks were often combined by gender-specific exposures: unassisted deliveries during attacks, displacement during pregnancy, and the trauma of losing husbands or primary earners.

Economic hardship further restricted healthcare access. Caregivers described their inability to afford transportation, medical fees, or medication – challenges also widely documented across Nigeria [51]. At the national level, Nigeria’s health system relies heavily on out-of-pocket expenditures, with households contributing more than 70% of total health spending. This financial model disproportionately affects poorer households and creates barriers even in peaceful settings [52].

Our findings illustrate how conflict intensifies these vulnerabilities. Insecurity inflates transportation costs, reduces livelihood opportunities, disrupts supply chains, and exacerbates household poverty. These effects were particularly gendered: women consistently described bearing the financial burden following the death, disappearance, or displacement of husbands. Limited access to independent income exacerbated their inability to seek care for themselves or their children. Men, in contrast, rarely reported personal economic strain, demonstrating a clear gender asymmetry in financial vulnerability.

### Community resilience and informal coping strategies

Despite profound challenges, caregivers demonstrated remarkable resilience through community-driven strategies. Vigilante groups, community patrols, youth escorts and initiatives such as the *No Boko* group provided security and logistical support, facilitating safer movement and access to healthcare. These findings align with literature emphasising the importance of local resilience in conflict-affected settings [53]. NGOs also played a key role by filling critical gaps left by weakened governmental services.

However, trust in formal health systems remained fragile. Recurrent conflict, inconsistent service delivery, and historical events such as the 2003 polio vaccine boycott [54] contributed to scepticism towards government-led health initiatives.

While the 2003 polio vaccine boycott is an important historical reference point, recent evidence shows that misinformation continues to influence vaccination attitudes across northern Nigeria. Rumours about infertility, vaccine safety, and government motives remain prevalent [13, 42, 55]. These concerns were echoed by participants in this study, indicating that despite improvements in immunisation outreach, longstanding sociopolitical mistrust persists.

Gender shaped vaccination attitudes in important ways. Women described highly personal consequences of misinformation - fear of infertility, pressure from husbands or in-laws, and scrutiny from extended family members. Patriarchal norms often meant that women´s access to vaccination services depended on male approval, reinforcing gatekeeping structures.

At the same time, community-based awareness campaigns – particularly those led by trusted religious and community leaders – were effective in rebuilding trust and encouraging vaccination uptake, as shown in other contexts [56]. The dissemination of accurate, accessible health information emerged as a key enabler of positive health-seeking behaviour [55].

Our findings show that resilience is rooted not only in physical or organisational infrastructures but also in social capital and informal problem-solving networks. These results resonate with broader research on community engagement in conflict-affected regions [56]. A scoping review of 19 studies on access to care in such contexts [57], emphasises that community-organised transport networks and grassroots collaboration enable continuity of care when formal systems fail [58]. Ager et al.’s study in Yobe State [58] similarly highlighted locally driven adaptations that sustain health service functionality despite insecurity.

Our study builds on this work by illustrating how these adaptive systems are interpreted and navigated at the household level. Caregivers’ decisions were shaped not only by available community solutions but also by perceptions of risk, trust, gendered obligations, and information asymmetries. We extend Ager et al.’s framework by showing that system-level resilience ultimately depends on micro-level decision-making processes within households.

### Gendered experiences of resilience

Just as insecurity and economic hardship were gendered, so too were coping mechanisms. Men framed their roles primarily around protection - participating in vigilante groups, escorting patients in emergencies and providing transportation. Women highlighted emotional strain, daily survival tasks and continuous demands of caring for children in conditions of instability. These differentiated experiences reinforce that community resilience is not uniform but stratified along gender lines.

### Intersections with behavioural theory

The relevance of these findings becomes clearer when viewed through established health behaviour models. The Health Services Utilization Model [59] helps explain how insecurity, distance, transport costs and medicine shortages functioned as major *perceived barriers* that often outweighed perceived benefits. Conflict heightened *perceived threats* associated not only with illness but with the act of travelling to health facilities. Within this environment, respected community actors - such as imams, youth group and local leaders - served as key *cues to action*, countering misinformation and encouraging caregivers to seek care.

The Theory of Planned Behavior (TPB) [60] further elucidates the impact of mistrust, gender norms and structural constraints. Negative attitudes towards government services, restrictive subjective norms influenced by patriarchal authority, and reduced behavioural control due to poverty and insecurity collectively shaped caregivers’ decisions. Conflict severely disrupted enabling resources such as transport, income and functioning health facilities, while community-led initiatives - vigilante escorts, informal transport networks - functioned as alternatives minimally restoring access to maternal and child health services.

In contrast to studies focusing primarily on access or health literacy as explanatory factors, our findings show how socio-political instability interacts with community networks, gender norms and household-level decision-making. Gender analysis demonstrates that conflict does not merely create barriers: it magnifies pre-existing inequities related to economic dependence, exposure to violence, and limited autonomy in healthcare decisions. This aligns with broader research on conflict-affected populations, where gender profoundly mediates access to resources, exposure to harm, and healthcare utilisation [13, 43, 44].

### Strengths and limitations

Our findings have several implications for policy and practice. First, rebuilding trust in health systems requires culturally sensitive interventions that respond to caregivers’ specific needs and concerns. Such strategies should include engaging community members, strengthening health infrastructures and ensuring consistent and reliable service delivery. Second, addressing economic barriers – through financial support, subsidised services, or transport assistance – has the potential to reduce inequalities and improve access to care. Third, strengthening health communication efforts, especially in underserved areas, is essential to counter misinformation and promote positive health behaviours.

However, several limitations of this qualitative study must be acknowledged. First, although nine FGDs were conducted across the BAY states, the experiences obtained from Borno, Yobe and Adamawa may not fully reflect the diversity of all conflict-affected populations in northeast Nigeria.

Second, although data saturation was achieved, it was assessed inductively through iterative team discussions rather than through a formalised saturation grid. Security constraints and logistical challenges common in conflict settings limited the opportunity to conduct additional confirmatory FGDs, which may have reduced the breadth of perspectives captured. Furthermore, the group-based nature of FGDs may have introduced social desirability bias or pressure to conform, potentially limiting open expression of negative or divergent views.

Third, FGDs were conducted in Hausa and later translated into English for analysis. Despite independent verification by bilingual researchers, subtle shifts in meaning, loss of nuance, or cultural misinterpretations cannot be entirely ruled out.

Fourth, data collection occurred in communities experiencing ongoing armed violence, which may have influenced participants’ willingness to disclose deeply personal or traumatic experiences. Despite careful facilitation and adherence to safety protocols, some individuals may have withheld information due to fear, discomfort, or perceived risk, potentially resulting in incomplete narratives.

Fifth, although the field research team consisted of Nigerian researchers familiar with local languages and cultural norms, the involvement of international researchers in the design, analysis, and interpretation phases may have introduced power dynamics or analytical biases.

Finally, participants’ displacement status—which can significantly shape access to services, exposure to violence, and the structure of community networks—was not systematically collected. This limits the study’s ability to account for important contextual variation within the conflict-affected population.

## Conclusion

This study provides critical insights into how intersecting factors - health-seeking behaviour, trust in health systems, exposure to conflict and vaccine hesitancy - shape caregivers’ decisions regarding child health services in conflict-affected northeast Nigeria. While structural and security-related barriers significantly restrict access to maternal and child services, communities continue to demonstrate resilience through informal support networks, community-led security arrangements and culturally grounded approaches to sharing health information.

Several actionable recommendations emerge for strengthening health system responses in these contexts. For the Nigerian government, improving maternal, newborn and child health (MNCH) in conflict-affected areas requires investment not only in service delivery but also in evidence-informed policymaking [61]. This includes institutionalising regular platforms in which policymakers, researchers, professional associations, and civil society jointly review emerging evidence and set priorities for MNCH programming. Existing evidence-to-policy mechanisms - such as national technical working groups and the Child Survival Action Plan - should be adequately resourced and mandated to translate research into updated guidelines, benefit packages, and primary healthcare standards [62].

For NGOs and humanitarian actors, expanding mobile outreach services, strengthening community-based health education, and partnering closely with trusted local leaders are essential for improving service uptake in insecure areas [63]. Programmes should explicitly address misinformation, gender-related barriers, and mobility constraints identified by caregivers, while also supporting community-driven resilience mechanisms that have proven effective during periods of insecurity.

Future research should examine geographical variation within conflict-affected settings, explore how trust in health systems evolves over time, and evaluate the effectiveness of community-driven resilience strategies. Methodologically, such work would benefit from longitudinal or mixed-methods designs, systematic attention to translation and cultural interpretation and triangulation of perspectives from caregivers, health workers, and policymakers. These approaches would enable a deeper understanding of dynamic changes in health-seeking behavior and generate more robust evidence for context-specific policy and programme development.

## Data Availability

No datasets were generated or analysed during the current study.
